# Multiple-Ion Releasing Bioactive Surface Pre-Reacted Glass-Ionomer (S-PRG) Filler: Innovative Technology for Dental Treatment and Care

**DOI:** 10.3390/jfb14040236

**Published:** 2023-04-21

**Authors:** Satoshi Imazato, Toshiyuki Nakatsuka, Haruaki Kitagawa, Jun-Ichi Sasaki, Satoshi Yamaguchi, Shuichi Ito, Hiroki Takeuchi, Ryota Nomura, Kazuhiko Nakano

**Affiliations:** 1 Department of Dental Biomaterials, Osaka University Graduate School of Dentistry, Suita 565-0871, Osaka, Japan; kitagawa.haruaki.dent@osaka-u.ac.jp (H.K.); sasaki.junichi.dent@osaka-u.ac.jp (J.-I.S.); yamaguchi.satoshi.dent@osaka-u.ac.jp (S.Y.); 2 Marketing Department, Shofu Inc., 11 Kamitakamatsu-cho, Fukuine, Higashiyama, Kyoto 605-0983, Kyoto, Japan; t-nakatsuka@shofu.co.jp; 3 Division of Clinical Cariology and Endodontology, Department of Oral Rehabilitation, School of Dentistry, Health Sciences University of Hokkaido, Ishikari 061-0293, Hokkaido, Japan; shu@hoku-iryo-u.ac.jp; 4 Department of Preventive Dentistry, Osaka University Graduate School of Dentistry, Suita 565-0871, Osaka, Japan; takeuchi.hiroki.dent@osaka-u.ac.jp; 5 Department of Pediatric Dentistry, Graduate School of Biomedical and Health Sciences, Hiroshima University, Hiroshima 734-8553, Hiroshima, Japan; rnomura@hiroshima-u.ac.jp; 6 Department of Pediatric Dentistry, Osaka University Graduate School of Dentistry, Suita 565-0871, Osaka, Japan; nakano.kazuhiko.dent@osaka-u.ac.jp

**Keywords:** dental, bioactive, ion release, glass filler, prevention, S-PRG

## Abstract

Surface Pre-Reacted Glass-ionomer (S-PRG) filler, which releases strontium (Sr^2+^), borate (BO_3_^3−^), fluoride (F^−^), sodium (Na^+^), silicate (SiO_3_^2−^), and aluminum (Al^3+^) ions at high concentrations, is a unique glass filler that are utilized in dentistry. Because of its multiple-ion releasing characteristics, S-PRG filler exhibits several bioactivities such as tooth strengthening, acid neutralization, promotion of mineralization, inhibition of bacteria and fungi, inhibition of matrix metalloproteinases, and enhancement of cell activity. Therefore, S-PRG filler per se and S-PRG filler-containing materials have the potential to be beneficial for various dental treatments and care. Those include restorative treatment, caries prevention/management, vital pulp therapy, endodontic treatment, prevention/treatment of periodontal disease, prevention of denture stomatitis, and perforation repair/root end filling. This review summarizes bioactive functions exhibited by S-PRG filler and its possible contribution to oral health.

## 1. Introduction

Patient-oriented dental treatment and care are considered essential. The philosophy of restorative treatments has shifted from restoration with aggressive cutting to less intervention to preserve sound tooth structure as much as possible. Based on the findings from many clinical studies, various self/professional care approaches have been gaining support, and patients can receive more customized oral care. To develop dental treatment and care further, novel technologies are needed, and bioactive materials are proposed as one such solution [1,2,3,4,5].

The definition of bioactive materials in dentistry has not been well established. Some researchers focus on functions that promote (re)mineralization or hard tissue formation [6]. However, in the life science field, bioactivity refers generally to the functions that positively interact with living cells and tissues. Therefore, bioactivity useful for restorative/preventive treatments and care are not so simple just to induce mineralization but can be more diverse to contribute to oral health as listed in Table 1. The mechanism for those effects can be based on biological, chemical, or mixed actions, as described in the policy statement for bioactive restorative materials proposed by FDI World Dental Federation [7]. 

Usage of particles that can release specific components is an effective method for achieving bioactive functions in dental materials [2,4,5]. For example, Surface Pre-Reacted Glass-ionomer (S-PRG) filler, a fine glass powder which releases multiple ions, is one of promising technologies. To date, a large number of in vitro and in vivo studies have reported the ability of S-PRG filler and S-PRG filler-containing materials to provide bioactive effects. In this review, bioactive functions exhibited by S-PRG filler and its benefits for various dental treatments and care are summarized. 

## 2. What Is S-PRG Filler

**Figure 1:**
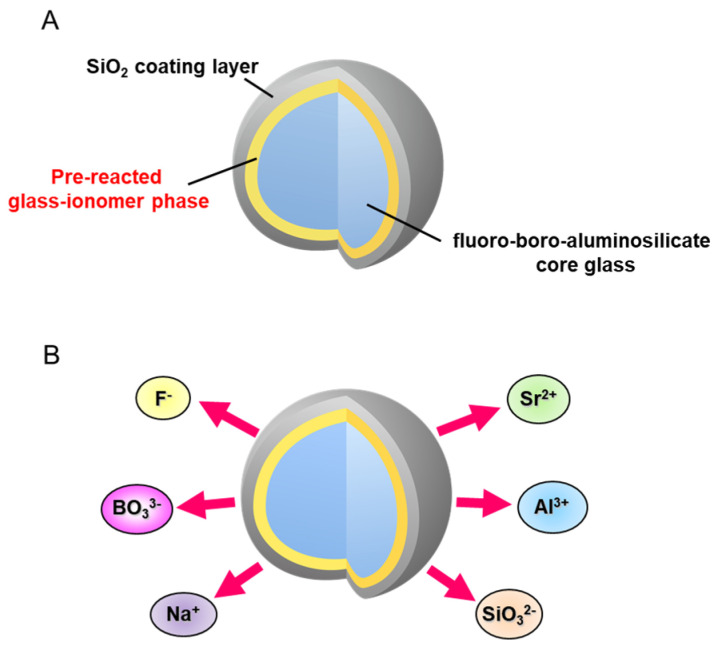
Surface Pre-Reacted Glass-ionomer (S-PRG) filler. (**A**) S-PRG filler is composed of three layers: outer SiO_2_ coating layer, pre-reacted glass-ionomer phase, and inner functional glass core. (**B**) S-PRG filler releases multiple ions: strontium (Sr^2+^), borate (BO_3_^3−^), fluoride (F^−^), sodium (Na^+^), silicate (SiO_3_^2−^), and aluminum (Al^3+^) ions.

S-PRG filler technology was developed by Shofu Inc. and the resin composites incorporating S-PRG filler were first commercialized in 2000. S-PRG filler is a three-layered fine glass particle composed of a SiO_2_ coating in the outermost layer followed by a pre-reacted glass-ionomer phase and a glass core (Figure 1A). The pre-reacted glass-ionomer phase is prepared by spraying a polyacrylic acid that penetrates the SiO_2_ coating layer and causes an acid-base reaction with the fluoro-boro-aluminosilicate core glass. Many products containing S-PRG filler are already available on the market, and a series of those materials are called GIOMER. Three types of S-PRG filler with particle sizes of 3.0, 0.8, and 0.4 μm are utilized for different materials among the GIOMER series. In addition to commercial products, S-PRG filler has been experimentally incorporated into a variety of materials.

**Figure 2:**
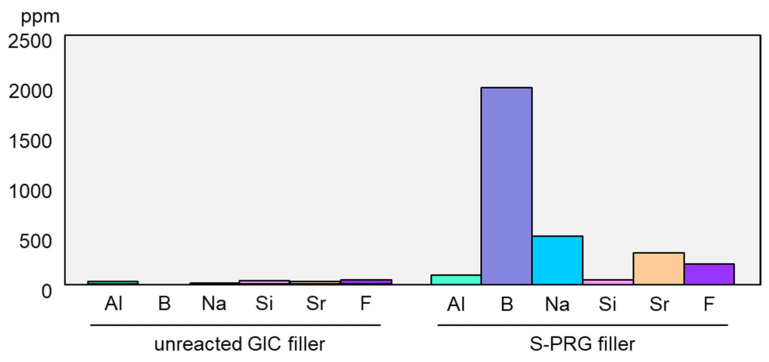
Comparison of ion release from S-PRG filler and conventional glass-ionomer filler. Release of ions from S-PRG filler or unreacted filler of conventional glass-ionomer cement was determined after stirring for 24 h in distilled water (mixing ratio of 1:1). The concentration of fluoride ions in the eluate was measured using a fluoride ion electrode, and those of other ions were measured using an inductively coupled plasma atomic emission spectrometer.

The pre-reacted glass-ionomer phase surrounding the glass core allows the S-PRG filler to release fluoride ions (F^−^). Additionally, because specially fabricated fluoro-boro-aluminosilicate glass is used as the core material, S-PRG filler releases five other ions: strontium (Sr^2+^), borate (BO_3_^3−^), sodium (Na^+^), silicate (SiO_3_^2−^), and aluminum (Al^3+^) ions (Figure 1B). The concentrations of the six ions released into water from S-PRG filler, especially BO_3_^3−^, Sr^2+^, and F^−^, are relatively high [8], much greater than those liberated from unreacted filler of conventional glass-ionomer cement (Figure 2). Notably, the six ions do not form salts and are found separately in the eluate.

## 3. Bioactive Functions Exhibited by S-PRG Filler

Owing to the release of multiple ions, S-PRG filler exhibits various bioactive functions, which are described below.

### 3.1. Tooth Strengthening

Ions released from S-PRG filler contribute to the strengthening of enamel and dentin, improving their acid resistance. It is well known that fluoride ions strengthen the tooth substrate through the formation of fluorapatite. The release of F^−^ from S-PRG filler at high concentrations is effective in providing such effects.

Uo et al. [9] revealed that the strontium content in enamel and dentin became 100 times greater after immersion in the eluate of S-PRG filler compared with that before immersion. It was further demonstrated that Al^3+^, BO_3_^3−^, and Sr^2+^ were taken up by enamel after 1 h of immersion in the eluate, and Sr showed remarkable incorporation of up to 7900 ppm after 28 days [10]. Strontium enhances the acid resistance of teeth by converting hydroxyapatite to strontium-apatite [11,12]. Uo et al. [9] investigated the local structure of Sr taken up by teeth using X-ray absorption fine structure analysis and determined that the structure of Sr in the enamel and dentin after immersion in the eluate of S-PRG filler was similar to that of synthetic Sr-incorporated hydroxyapatite, suggesting that Sr released from S-PRG filler can be incorporated into the Ca site of hydroxyapatite.

Hiraishi et al. [13] examined the interaction of borate ions released from S-PRG filler with the apatite of enamel and dentin and revealed that the borate ion adsorbed in the enamel and dentin was in a tetra-coordinated form, which possesses a buffer capacity, helps protect the tooth structure against acid attacks, and promotes remineralization.

### 3.2. Acid Neutralization

S-PRG filler modulates the pH of the surrounding medium and shifts it toward neutral and weak alkaline values [14]. Although further investigations are required to clarify the acid-buffering mechanism, the released Sr^2+^ and Na^+^ are considered to promote acid buffering. 

### 3.3. Promotion of Mineralization

**Figure 3:**
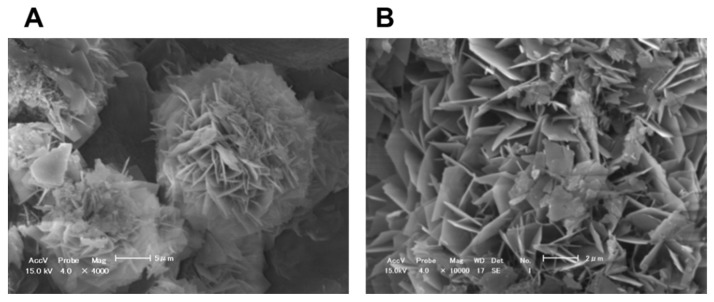
Mineral induction by eluate of S-PRG filler-containing adhesive. (**A**) Globular deposit formed in the presence of eluate of S-PRG filler-containing adhesive (Shake One). (**B**) Control. The eluate of S-PRG filler-containing adhesive promoted mineral formation on phosvitin-immobilized agarose beads placed in mineralizing solution consisting of 2.24 mM CaCl_2_, 1.34 mM KPO_4_, 150 mM KCl, and 10 mM Hepes at pH 7.4.

The S-PRG filler eluate containing Sr^2+^ at high concentrations can react with other ions in mineralizable solutions to induce chemical mineralization. The eluate obtained from S-PRG filler-containing materials promotes mineral deposit formation on the surface of phosvitin-immobilized agarose beads (model of demineralized dentin) placed in the mineralizing solution [8] (Figure 3). By micro-area X-ray diffraction analysis, the minerals that formed with a globular structure were determined to be apatite.

Nemoto et al. [15] reported that the eluate of S-PRG filler upregulated the mRNA expression level of the osteogenic differentiation marker (i.e., alkaline phosphatase) in human bone marrow-derived stromal cells. As a consequence, the S-PRG filler eluate promoted the mineralization of the extracellular matrix [15]. Ishigure et al. [16] reported that the eluate of S-PRG filler promoted the proliferation of dental pulp stem cells and enhanced their alkane phosphatase activity. These results suggest that S-PRG filler can biologically promote apatite formation and hard tissue formation, as well as chemically. 

### 3.4. Inhibition of Bacteria and Fungi

Numerous studies have demonstrated antibacterial effects of the S-PRG filler eluate against bacteria in human saliva and dental plaque, such as streptococci, *Enterococcus faecalis*, *Actinomyces israelii*, *Propionibacterium acnes*, *Porphyromonas gingivalis*, and *Fusobacterium nucleatum* [17].

Nomura et al. [18] revealed that the S-PRG filler eluate inhibited *Streptococcus mutans* growth in a dose-dependent manner, especially before the logarithmic growth phase. The growth of *S. mutans* in the absence of S-PRG filler eluate reached a stationary phase 7 h after incubation, whereas the time for *S. mutans* growth was extended in the presence of S-PRG filler eluate. The ions mainly responsible for such effects are considered to be BO_3_^3−^ and F^−^. Borate ions have been used as a preservative in the ophthalmic field, and fluoride ions at high concentrations are also known to inhibit bacteria. Miki et al. [19] investigated *S. mutans* growth in the presence of Na^+^, SiO_3_^2−^, Sr^2+^, BO_3_^3−^, Al^3+^, or F^−^ at the concentrations released from the experimental resin composites containing S-PRG filler at 55.9 vol% and determined that BO_3_^3−^ and F^−^ significantly inhibited the growth.

**Figure 4:**
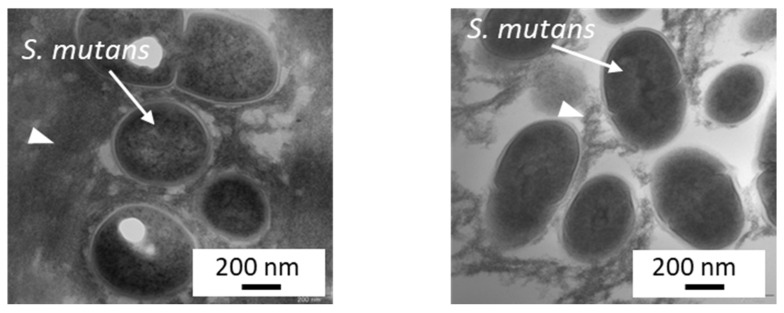
Inhibition of *S. mutans* glucan synthesis by S-PRG filler eluate. *S. mutans* MT8148 (1.0 × 10^7^ CFU/mL) was incubated for 18 h at 37 °C with the addition of 1% sucrose without (**left**) or with (**right**) the 25% S-PRG filler eluate. Arrow: *S. mutans* cell, Arrowhead: glucan formed.

**Figure 5:**
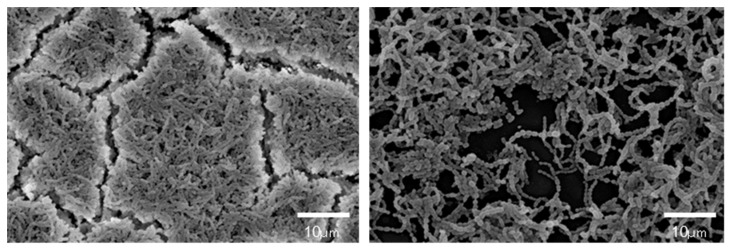
Inhibition of biofilm formation by S-PRG filler eluate. *S. mutans* NCTC10449 suspension supplemented with 1% sucrose was incubated for 24 h without (**left**) or with the 20% S-PRG filler eluate (**right**). In the presence of the 20% S-PRG filler eluate, *S. mutans* biofilm formation was inhibited.

Kitagawa et al. [20] reported that even low concentrations of BO_3_^3−^ or F^−^, at which bacterial growth was not affected, can inhibit *S. mutans* glucose metabolism and acid production. A detailed study conducted by Nomura et al. [18] clarified that the eluate of S-PRG filler prominently downregulated operons related to *S. mutans* sugar metabolism, such as the pdh operon encoding the pyruvate dehydrogenase complex and the glg operon encoding a putative glycogen synthase. According to such effects, when cultured with the 20–25% S-PRG filler eluate, glucan synthesis by *S. mutans* was inhibited (Figure 4), and the biofilm formed was determined to be sparse, with less extracellular matrix (Figure 5).

Ions released from S-PRG filler are effective in inhibiting bacteria related to periodontal disease and suppressing their pathogenic factors. It was reported that the eluate of S-PRG filler inhibited the growth of *P. gingivalis*, *F. nucleatum*, and *Aggregatibacter actinomycetemcomitans* [21], and suppressed the hemagglutination and gingipain activity of *P. gingivalis* [21,22]. It was also determined that the coaggregation of *P. gingivalis* and *F. nucleatum*, essential for subgingival biofilm formation, can be prevented in the presence of S-PRG filler eluate [22]. 

Tamura et al. [23] reported that the eluate of S-PRG filler decreased the amount of hydrogen peroxide and catalase activity in *Candida albicans*. Furthermore, the S-PRG filler eluate prevented the growth and biofilm formation of *C. albicans* and inhibited their dimorphism conversion. The eluate of S-PRG filler also suppressed the activity to produce secreted aspartyl proteinases, which are major pathogenic factors of *C. albicans*.

### 3.5. Inhibition of Matrix Metalloproteinases

Dentin matrix metalloproteinases (MMP) are a family of host-derived proteolytic enzymes that are trapped within the dentin matrix and can hydrolyze the organic matrix of dentin. MMPs break down collagen at the resin–dentin interface and compromise the durability of dentin bonding. Therefore, many reports are available on application of MMP inhibitors during bonding procedures to improve the long-term stability of dentin bonding. The S-PRG filler eluate inhibits the activity of collagen-bound MMPs and dentin matrix degradation, similar to 2% chlorhexidine digluconate, which is known as a MMP inhibitor [24]. Salim et al. [25] also reported that the eluate of S-PRG filler showed a reduction in MMP activity and revealed that F^−^ ions most effectively inhibited the enzymatic activity among the six ions released from S-PRG filler. 

### 3.6. Enhancement of Cell Activity

Yamaguchi-Ueda et al. [26] examined the effects of various concentrations of S-PRG filler eluates on the growth and migration of gingival fibroblasts. They revealed that multiple ions present in the S-PRG filler eluate can stimulate migration of human gingival fibroblast cells via upregulation of the extracellular signal-regulated kinase 1/2 (ERK 1/2) signaling pathway. This finding indicates that the ions in the S-PRG filler eluate promoted cell activity, which may assist in oral wound healing. 

Takeuchi et al. [27] reported that the eluate of S-PRG filler inhibited penetration of virulence factors of *P. gingivalis*, such as lipopolysaccharide and peptidoglycan, into gingival epithelial cells. The eluate of S-PRG filler induced the expression of coxsackievirus and adenovirus receptor (CXADR), a tight barrier of gingival epithelium, in immortalized human gingival epithelial cells, indicating that the S-PRG filler eluate enhanced the epithelial barrier function.

## 4. Benefits of S-PRG Filler for Dental Treatment and Care

Many products containing S-PRG filler are commercially available, such as resin composites, adhesives, resin cements, coating resins, fissure sealants, polishing pastes, and temporary fillings (Table 2). Additionally, S-PRG filler has been incorporated experimentally into inorganic cements, root canal sealers, denture bases, tissue conditioners, denture adhesives, toothpastes, varnishes, CAD/CAM composites, and toothbrush filaments. Even when S-PRG filler is incorporated into resin-based materials with silanization, multiple ions are released at high concentrations. The capacity for releasing six ions (i.e., Sr^2+^, BO_3_^3−^, F^−^, Na^+^, SiO_3_^2−^, and Al^3+^) endows the S-PRG filler with various therapeutic effects, which are useful for restorative treatment, caries prevention/management, vital pulp therapy, endodontic treatment, prevention/treatment of periodontal disease, prevention of denture stomatitis, and perforation repair/root end filling. In this regard, S-PRG filler-containing materials differ from conventional ones that only release ions to promote remineralization, such as glass-ionomer cements or fluoride-releasing resin composites.

### 4.1. Restorative Treatment

To develop restorative treatments with preventive effects, S-PRG filler has been incorporated in resin composites, adhesives, and resin cements. 

A ligand exchange mechanism within the glass-ionomer phase offers S-PRG filler the ability to release and recharge fluoride ions. The proprietary resin composites containing S-PRG filler (Beautifil II, Shofu Inc., Kyoto, Japan) release high concentrations of fluoride ions and can be recharged by exposure to 5000 ppm sodium fluoride solution for 5 min [28].

**Figure 6:**
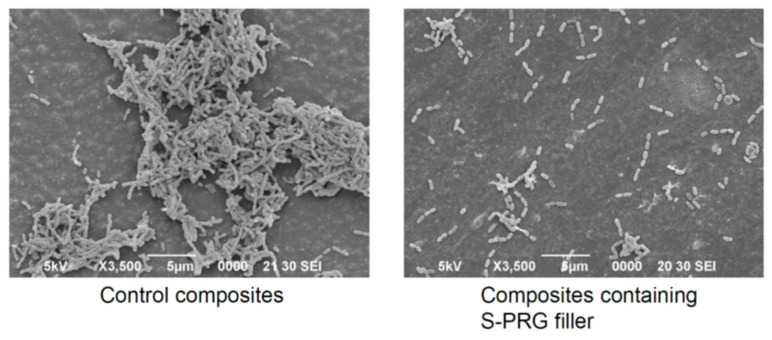
Inhibitory effects of *S. mutans* biofilm formation on resin composites containing S-PRG filler. Reproduced with permission from Imazato et al. [4]. Copyright: Japanese Society for Dental Materials and Devices.

**Figure 7:**
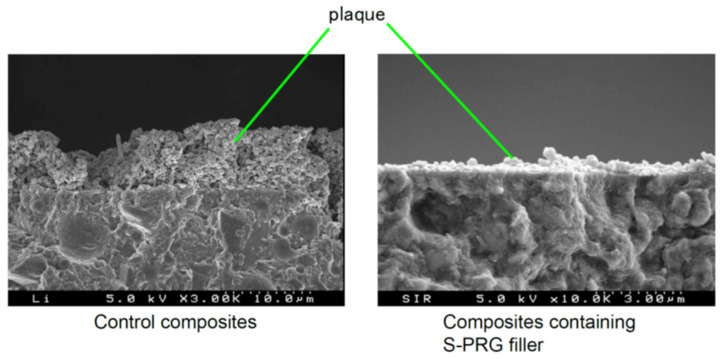
Plaque accumulated on resin composites after placement in a human mouth for 24 h. Reproduced with permission from Imazato et al. [4]. Copyright: Japanese Society for Dental Materials and Devices.

Several studies have demonstrated that resin composites containing S-PRG filler exhibit antibacterial effects against oral bacteria [4,5]. Miki et al. [19] examined the inhibitory effects of experimental resin composites containing different ratios of S-PRG filler on *S. mutans* growth and reported that the specimens containing S-PRG filler at 28.0 vol% or greater inhibited bacterial growth on their surfaces. They further demonstrated that the inhibitory effects were mainly attributed to the release of BO_3_^3−^ and F^−^. It was also reported that the eluate of S-PRG filler suppressed the adherence of *S. mutans* [22], and thus, *S. mutans* biofilm formation was inhibited on the surface of resin composites containing S-PRG filler (Figure 6). An in situ study assessing plaque accumulation on resin composites placed in human mouths for 24 h showed significant suppression of dental plaque maturation on the surface of resin composites containing S-PRG filler [29] (Figure 7). 

An investigation of demineralization in the tooth structure surrounding filling materials revealed that S-PRG filler-containing resin composites inhibited the demineralization of wall and outer lesions in enamel and dentin and increased the surrounding tooth hardness [30]. Lai et al. [31] investigated the anti-demineralization effects of restorations using S-PRG filler-containing resin composites (Beautifil II) and adhesives (FL-Bond II) on the root surfaces. Using an oral biofilm reactor and pH cycling artificial caries model, they demonstrated that the combination of Beautifil II and FL-Bond II exhibited a cumulative effect to inhibit demineralization of root dentin adjacent to restoration. 

It has been reported that *S. mutans* contains esterases that can potentially degrade resin-based restorative materials [32]. Gautam et al. [33] revealed by in vitro test that degradation by *S. mutans* was reduced for resin composites containing S-PRG filler (Beautifil II) compared with other commercial resin composites. On the other hand, Yoshihara et al. [34] reported production of many holes on the surface of S-PRG filler-containing resin composites after immersion in lactic acid at pH 4.0 for 3 days, while no change in surface integrity was detected for other conventional resin composites. They described that such surface alteration was caused by dissolution of S-PRG filler. Although several clinical studies revealed their excellent performance [35,36], long-term surface durability of S-PRG filler-containing resin composites remains to be investigated in detail.

Recently, CAD/CAM resin composite blocks containing S-PRG filler have been developed for the restoration of primary molar teeth. Nakase et al. [37] reported that S-PRG filler-containing CAD/CAM resin composite blocks demonstrated acceptable physical properties and wear performance for clinical use. Moreover, an S-PRG filler-containing CAD/CAM resin composite crown is advantageous for primary tooth restoration because it can be cemented using a conventional glass-ionomer cement through the chelation reaction between Sr and polycarboxylic acid [38].

### 4.2. Caries Prevention/Management

The S-PRG filler modulates the pH of the surrounding medium and shifts it toward neutral and weak alkaline values [14]. Additionally, the release of F^−^ and Sr^2+^ from S-PRG filler strengthens the tooth substrate, as described above. These effects are beneficial for the prevention and management of caries. Many studies have demonstrated that ions released from S-PRG filler in various materials are taken up by adjacent enamel and dentin to prevent demineralization, including adhesives [39], endodontic sealers [40], orthodontic adhesives [41], pit/fissure sealants [42], coating materials [43,44], resinous vanishes [45], and denture base resins [46].

A resinous coating material containing S-PRG filler (PRG Barrier Coat, Shofu Inc.) protects the enamel surface from demineralization caused by acidic attack [44,47]. Ions released from S-PRG filler in PRG Barrier Coat exert acid-neutralizing effects near the coated surface. Uptake of F and Sr released from PRG Barrier Coat by the tooth substrate can be effective to inhibit demineralization [48]. Ma at al. [43] demonstrated that PRG Barrier Coat containing S-PRG filler can protect root surfaces from demineralization by producing a coating layer with a thickness of approximately 200 µm. It was also shown that the pH drop in *S. mutans* clumps caused by glucose on the surface of the PRG Barrier Coat was reduced, possibly because the glucose metabolism was hindered by the released ions as well as acid buffering [43].

The incorporation of S-PRG filler into a resin-based pit and fissure sealant provides acid neutralization capacity without deteriorating the sealing effectiveness [49,50] or the ability to release and recharge fluoride ions [51]. Some clinical studies demonstrated caries preventive effects of commercially available S-PRG filler-containing pit/fissure sealant (BeautiSealant, Shofu Inc.) [52,53]. Penha et al. [53] conducted a randomized clinical trial to compare the evolution of caries in newly erupted permanent molars and determined that the S-PRG sealant group presented higher sound teeth predominance compared with another fluoride-releasing sealant after 12 months.

A polishing paste containing S-PRG filler, including a commercial one containing 5% S-PRG filler (PRG Pro-Care Gel, Shofu Inc.), is useful for controlling caries incidence and remineralization. Iijima et al. [54] reported that human enamel polished with S-PRG filler-containing paste, following immersion in the demineralizing solution at pH 4.5, exhibited greater surface hardness and smoothness than those polished with fluoridated paste or nano-hydroxyapatite-containing paste. Furthermore, the surface hardness of demineralized dentin polished with the S-PRG filler-containing paste recovered after immersion in mineralizing solution for 1 month, indicating that the S-PRG filler-containing paste can promote dentin remineralization [55]. A recent study revealed that PRG Pro-Care Gel has a similar potential to remineralize the demineralized root dentin as a 38% silver diamine fluoride solution [56].

Amaechi et al. [57] examined the effectiveness of experimental toothpastes containing S-PRG filler in preventing tooth surface demineralization using Featherstone’s pH cycling model to develop early caries. It was determined that S-PRG filler-containing dentifrice was more effective in preventing tooth demineralization than 1100 ppm F-containing toothpaste, highlighting the potential of S-PRG filler to assist individuals at high risk of developing caries [58]. The potential of S-PRG filler-containing toothpaste to remineralize the demineralized enamel surface has also been shown in situ [59]. Experimental toothpaste containing 5% S-PRG filler showed remineralizing effects similar to those of toothpaste containing 1500 ppm F, even showing greater activity to recover mineral loss in the subsurface regions.

Moecke et al. [60] investigated the remineralizing effects of experimental varnishes containing S-PRG filler. They revealed that the varnish containing 40% S-PRG filler more effectively promoted the remineralization of demineralized bovine enamel compared with the varnish containing 5% sodium fluoride.

### 4.3. Vital Pulp Therapy

S-PRG filler can promote tertiary dentinogenesis. As pulp capping materials, several attempts have been made to incorporate S-PRG filler into adhesives [61,62], self-adhesive resins [63], and inorganic cements [64,65].

Experimental inorganic cement, prepared by mixing S-PRG filler with copolymers of acrylic acid and tricarboxylic acid, induces complete tertiary dentin formation at 2 and 4 weeks after pulp exposure in rat molars [64]. Strontium released from S-PRG cement was determined to transfer into the pulpal tissue and contribute to the induction of mineralization and dentinogenesis [65]. These functions are based on biological action because the S-PRG cement enhanced the expression of genes related to osteo/dentinogenic differentiation, such as *CXCL-12* and *TGF-β1*, which contribute to tertiary dentin formation during the healing process in pulpal tissue [65].

It has been reported that lithium ions can activate the Wnt/β-catenin signaling pathway and induce dentin formation in pulpotomized teeth [66]. Therefore, trials to add LiCl to S-PRG cement were conducted, revealing that reparative dentin formation in rat teeth was enhanced through activation of the Wnt/β-catenin canonical signaling pathway without adversely affecting the sealing property of the cement [67,68].

### 4.4. Endodontic Treatment

Apical periodontitis is an infectious disease, and the treatment requires careful bacterial eradication inside filled root canals. However, the complete elimination of bacterial infection in root canals is difficult to achieve using only mechanical instrumentation, irrigation, and medication. Therefore, endodontic filling materials capable of eliminating residual bacteria inside root canals have been proposed as a possible solution to this problem. An experimental zinc oxide-based endodontic sealer containing S-PRG filler demonstrated the sustained release of multiple ions (i.e., Sr^2+^, BO_3_^3−^, F^−^, Na^+^, SiO_3_^2−^, and Al^3+^) [40] and antibacterial effects against *E. faecalis* and *P. gingivalis* [69,70].

The eluate obtained from experimental root canal sealer containing S-PRG filler was reported to downregulate mRNA expression levels of proinflammatory cytokines, such as interleukin (IL)-1α, IL-6, and TNF-α, in LPS-stimulated RAW264.7 cells, suggesting its anti-inflammatory effects [71]. Moreover, an experimental sealer containing S-PRG filler can promote the healing of periapical lesions in vivo [70,72,73].

### 4.5. Prevention/Treatment of Periodontal Disease

**Figure 8:**
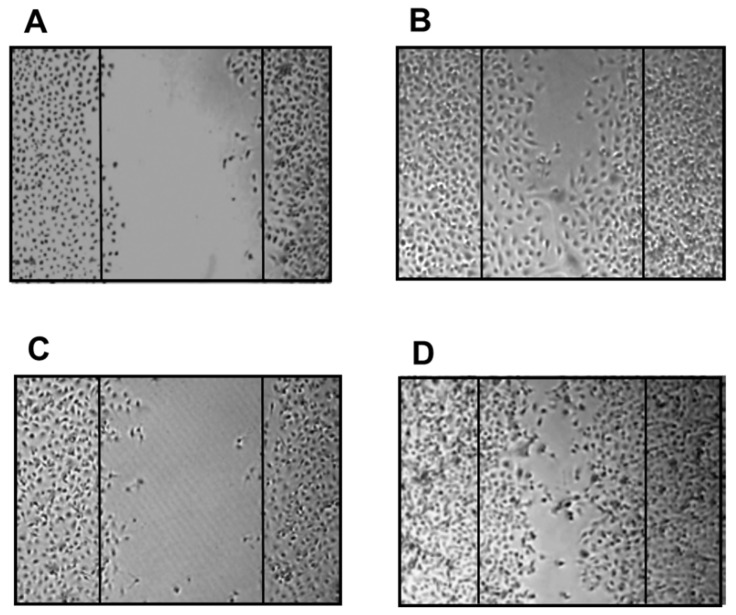
Recovery of migration activity in epithelial cells by S-PRG filler eluate. Human gingival epithelial cells were infected with *P. gingivalis*, and their migration was assessed after 24 h of culture. (**A**) Before culture, (**B**) after culture without *P. gingivalis* infection, (**C**) after culture with *P. gingivalis* infection, and (**D**) after culture with *P. gingivalis* infection and 1% S-PRG filler eluate.

The S-PRG filler eluate suppresses the activity of periodontitis-related bacteria [21,22] and inhibits penetration of *P. gingivalis* virulence factors into gingival epithelial cells [27]. It also promotes recovery of migration activity in epithelial cells infected with *P. gingivalis* (Figure 8). In recent years, several animal studies have verified the ability of S-PRG filler to help prevent the progression of periodontal disease. Iwamatsu-Kobayashi et al. [74] investigated the effects against tissue destruction induced in a mouse periodontal disease model and revealed that periodical dripping of the S-PRG filler eluate suppressed the bone loss and resulted in greater volume and density of alveolar bone. It has also been reported that ultrasonic irrigation with S-PRG filler dispersion can improve periodontal parameters, such as gingival index, bleeding on probing, probing pocket depth, and clinical attachment level when applied to a periodontal defect in a dog [75,76]. Irrigation with S-PRG filler dispersion reduced the ratio of red complex (i.e., *Tannerella forsythia*, *P. gingivalis*, and *Treponema denticola*) among the bacterial flora in the periodontal pocket [76].

### 4.6. Prevention of Denture Stomatitis

A denture base with antifungal effects may be useful for the prevention of denture stomatitis. The morphology of *C. albicans* cells remains in the yeast form in a normal resident flora, but it grows from the yeast form to the hyphal form by creating a longer germ tube when grown in an inflammatory environment in a human host [77]. The eluate of S-PRG filler prevents adherence of *C. albicans* to denture base resin and inhibits the dimorphism conversion of *C. albicans* [23]. The incorporation of S-PRG filler into polymethyl methacrylate-based resin effectively inhibited biofilm formation by *C. albicans*, with reduced length of the hyphae [78]. Similar results have been reported for the addition of S-PRG filler to other materials in the field of removable prosthodontics, such as tissue conditioners [79,80] and poly(methyl vinyl ether-alt-maleic anhydride)-based denture adhesives [81]. The multiple-ion release characteristics of S-PRG filler may contribute to the oral health of the elderly population by preventing the onset of oral candidiasis.

### 4.7. Perforation Repair/Root End Filling

**Figure 9:**
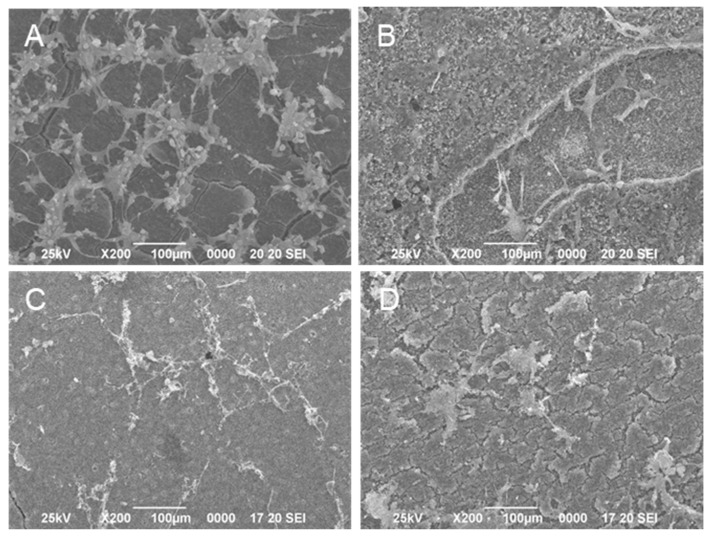
Proliferation of osteoblastic cells on the material surface. (**A**) Experimental S-PRG cement, (**B**) calcium silicate-based cement (ProRoot MTA), (**C**) reinforced zinc oxide cement, and (**D**) glass-ionomer cement. MC3T3-E1 cells were seeded on the set specimen and cultured for 24 h.

Hirata-Tsuchiya et al. [72] reported that the application of commercially available resin composites containing S-PRG filler for sealing root perforation can facilitate periodontal tissue healing and support good clinical outcomes. Inorganic cement containing S-PRG filler exhibits excellent cytocompatibility for osteoblastic cells, similar to a calcium silicate-based cement (Figure 9). Considering the enhancement of cell activity by released ions, S-PRG filler-containing materials may be useful for perforation repair and root end filling, for which calcium silicate-based cements have been widely used.

## 5. Ion Release Profile of S-PRG Filler-Containing Materials

**Figure 10:**
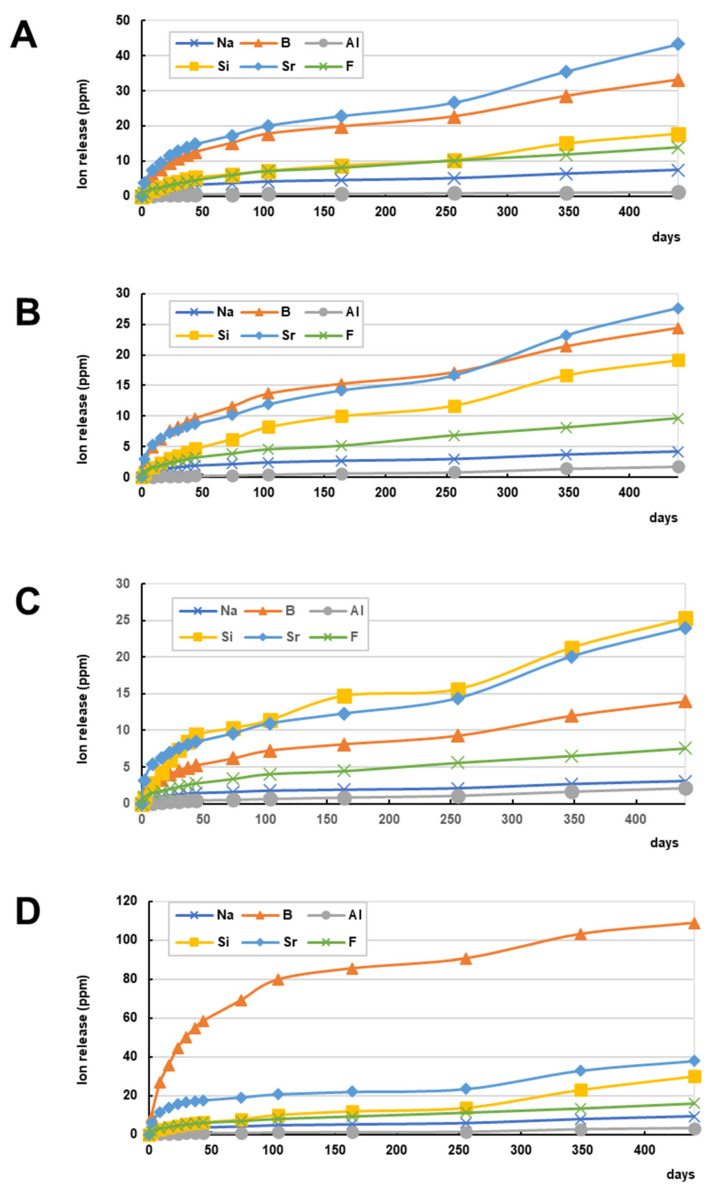
Ion release profile of commercially available S-PRG filler-containing restorative materials. Cumulative concentrations of ions released from (**A**) resin composites (Beautifil II), (**B**) flowable-type resin composites (Beautifil Flow Plus), (**C**) resin cement for luting (ResiCem EX), and (**D**) bonding agent in a two-step self-etch adhesive (FL-Bond II). The disc-shaped cured specimen (15 mm diameter, 1 mm thickness) was immersed in 5 mL of distilled water, which was replaced periodically. The concentration of fluoride ions in the eluate was measured using a fluoride ion electrode, and those of other ions were measured using an inductively coupled plasma atomic emission spectrometer.

To provide clinical benefits for restorative treatments, sustained or controlled release of active components is essential for agent-releasing bioactive materials. Figure 10 shows the release profile of six ions from commercially available S-PRG filler-containing resin composites, an S-PRG filler-containing resin cement for luting, and an S-PRG filler-containing bonding agent in a two-step self-etch adhesive. All materials demonstrated continuous release of six ions for up to 440 days, with the release of Sr^2+^ and BO_3_^3−^ at high concentrations for the resin composites and the resin cement. The bonding agent containing S-PRG filler exhibited a different release pattern and released a large amount of BO_3_^3−^. Continuous release of fluoride for 440 days was confirmed for all restorative materials.

**Figure 11:**
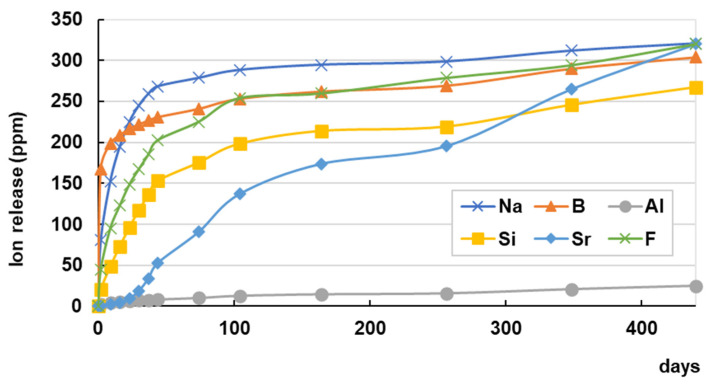
Ion release profile of coating resin containing S-PRG filler. Cumulative concentrations of ions released from commercial coating resin containing S-PRG filler (PRG Barrier Coat). The disc-shaped cured specimen (15 mm diameter, 1 mm thickness) was immersed in 5 mL of distilled water, which was replaced periodically. The concentration of fluoride ions in the eluate was measured using a fluoride ion electrode, and those of other ions were measured using an inductively coupled plasma atomic emission spectrometer.

The ion release profile of a coating resin containing S-PRG filler is depicted in Figure 11. The concentrations of all ions released from the coating resin were much greater than those released from the restorative materials, and a large amount was released within 100 days, except for Sr^2+^, which showed relatively constant release pattern.

## 6. Conclusions

A large number of in vitro and in vivo (animal) studies have been conducted to assess the bioactive functions exhibited by S-PRG filler and S-PRG filler-containing materials. In addition to commercial products containing S-PRG filler, many reports on experimental materials are available. The S-PRG filler has the potential as a bioactive material to contribute to the success of various dental treatments and care. The main concern is that a limited number of clinical studies on the benefits of bioactive functions exhibited by S-PRG filler and S-PRG filler containing materials is available. Most of the evidence described in this review is based on in vitro or animal studies. Although several studies evaluated long-term action of ion-release from S-PRG filler, it is well known that the bench-to-clinic translation is not so simple. Future research in clinical settings and their accumulation are expected to elucidate further the practicality and efficacy of bioactive S-PRG filler for oral health promotion.

## Figures and Tables

**Table 1:** Bioactive functions for restorative/preventive treatments and care.

Promotion of mineralization/hard tissue formation
Control of bacterial infection
Prevention of inflammation
Promotion of tissue regeneration

**Table 2:** Commercially available materials containing S-PRG filler.

Materials	Type	Product Name
Resin composites	Packable	BEAUTIFIL II BEAUTIFIL II LS BEAUTIFIL Next * BEAUTIFIL Enamel BEAUTIFIL Gingiva BEAUTIFIL-Bulk Restorative BEAUTIFIL e Posterior BEAUTIFIL Unishade *
	Flowable	BEAUTIFIL Flow BEAUTIFIL Flow Plus BEAUTIFIL Flow Plus X BEAUTIFIL-Bulk Flowable BEAUTIFIL Injectable BEAUTIFIL Injectable X BEAUTIFIL Unishade Flow * FIT SA BEAUTIFIL Kids * BEAUTIFIL Kids SA
	Core build up	BeautiCore Flow Paste * BeautiCore LC *
Adhesives	2-step self-etch	FL-Bond II
	1-step self-etch	Fluoro Bond Shake One *
Resin cements	With adhesive	ResiCem EX *
	Self-adhesive	BeautiCem SA
	Veneer cement	BeautiCem Veneer
	Orthodonitcs	BeautiOrtho Bond II *
Teeth coating resins	Preventive	PRG Barrier Coat
	Esthetic	BeautiCoat
Fissure sealants		BeautiSealant
Cleaning/polishing pastes	For tooth surface	PRG Pro-Care Gel α
	For composites	PRG CompoGloss
Temporary fillings	Fillings	PRG Protect Seal *
	Cementing	IP Temp Cement *

* only available on Japanese market.

## Data Availability

The data that support the findings of this study are available from the corresponding author upon reasonable request.
